# BDNF/TRKB axis provokes EMT progression to induce cell aggressiveness via crosstalk with cancer-associated fibroblasts in human parotid gland cancer

**DOI:** 10.1038/s41598-022-22377-9

**Published:** 2022-10-20

**Authors:** Kazumasa Moriwaki, Masaki Wada, Hiroko Kuwabara, Yusuke Ayani, Tetsuya Terada, Masaaki Higashino, Ryo Kawata, Michio Asahi

**Affiliations:** 1 Department of Pharmacology, Faculty of Medicine, Osaka Medical and Pharmaceutical University, 2-7, Daigaku-Machi, Takatsuki, Osaka 569-8686 Japan; 2 Department of Otolaryngology-Head and Neck Surgery, Faculty of Medicine, Osaka Medical and Pharmaceutical University, 2-7, Daigaku-Machi, Takatsuki, Osaka 569-8686 Japan; 3 Department of Pathology, Faculty of Medicine, Osaka Medical and Pharmaceutical University, 2-7, Daigaku-Machi, Takatsuki, Osaka 569-8686 Japan

**Keywords:** Cancer, Cell biology, Oncology

## Abstract

Parotid gland cancer (PGC) is a rare malignancy and its molecular characteristics remain poorly understood, which has precluded the development of effective drug therapies. Given the poor prognosis of many human cancers in which tropomyosin receptor kinase B (TRKB) is highly expressed, we investigated the involvement of brain-derived neurotrophic factor (BDNF)/TRKB pathway in PGC cells using clinical specimens and observed upregulation of TRKB and BDNF. In primary culture systems of patient-derived PGC cells and cancer-associated fibroblasts (CAFs), PGC cells co-cultured with CAFs exhibited significant upregulation of BDNF and epithelial-mesenchymal transition (EMT). Similar results were observed in PGC cells treated with conditioned medium from co-cultures of PGC cells with CAFs. Administration of TRK inhibitors suppressed BDNF-induced cell migration in PGC cells. Immunohistochemical and clinicopathological analyses of tumors from patients with PGC revealed that BDNF and TRKB were highly expressed in both tumor cells and stromal cells such as CAFs, and TRKB expression levels in PGC cells were significantly correlated with aggressive features, including vascular invasion, nodal metastasis, and poor prognosis. Collectively, these data suggest that the BDNF/TRKB pathway regulates PGC cell aggressiveness via crosstalk with CAFs and is a potential therapeutic target for PGC harboring invasive and metastatic features.

## Introduction

Parotid gland cancer (PGC) is a rare malignancy in head and neck tumors, accounting for approximately 0.5% of all malignancies. PGC comprises a large variety of histological subtypes, and the expression levels of several key proteins are important features for predicting the prognosis and therapeutic strategies for PGC^1–3^. However, the molecular features of PGC are poorly understood, which has precluded effective diagnosis and individualized drug therapies. Indeed, PGC remains a global public health issue.

The tropomyosin receptor kinase (TRK) family consists of three tyrosine kinase receptors: TRKA, TRKB, and TRKC, which are encoded by *NTRK1*, *NTRK2*, and *NTRK3* genes, respectively. TRK proteins play a pivotal role in neuronal development and maintenance as well as in disease processes via activation of downstream signaling cascades of neurotrophin receptors^4^. Aberrant TRK signaling is observed in various human malignancies, and the TRK family is emerging as a critical target for anticancer therapies^5–8^. TRKB is one of the most well-documented members of the TRK family. TRKB and its ligand, brain-derived neurotrophic factor (BDNF), are highly expressed and associated with poor survival in various cancer types, including breast cancer^9^, colon cancer^10^, lung cancer^11^, pancreatic cancer^12^, ovarian cancer^13^, and oral squamous cell carcinoma (OSCC)^14^. Upregulation of the BDNF/TRKB pathway in cancer cells promotes cell proliferation and survival via the RAS/MAPK, PI3K/PDK1/AKT, and PLCγ pathways, and induces anoikis suppression and epithelial-mesenchymal transition (EMT) via the induction of Zeb1, Twist, and/or Snail^15–18^. Murine transplantation models of tumor cells have demonstrated that TRKB promotes metastasis in several types of tumors, including lung adenocarcinoma^11^, breast cancer^9^, and neuroblastoma^19^. EMT is regulated by tumor stromal cells and constitutes a crucial step during all stages of cancer progression, especially tumor invasion, dissemination, and metastasis to colonization^20, 21^. Among stromal cells, cancer-associated fibroblasts (CAFs) are critical for EMT and directly or indirectly support tumor growth, invasion, and metastasis by secreting growth factors and inflammatory mediators such as TGF-β, HGF, FGF, and IL-6^22^. CAFs also secrete BDNF to regulate cancer progression in some types of cancer^23, 24^. Nevertheless, the roles of the BDNF/TRKB pathway in PGC cells and its interaction with CAFs remain poorly understood.

In this study, we investigated the involvement of the BDNF/TRKB pathway in PGC. Using primary co-culture systems and immunohistochemical and clinicopathological analyses of human samples from patients with PGC, we demonstrate that upregulation of the BDNF/TRKB pathway in PGC cells regulates PGC cell aggressiveness via crosstalk with CAFs. Our findings highlight the BDNF/TRKB pathway as a therapeutic target for PGC harboring invasive and metastatic features.

## Methods

### Patients and tissue specimens

Parotic tumor tissues and adjacent normal tissues were obtained from 23 patients diagnosed with PGC (adenoid cystic carcinoma, 52.2%; salivary duct carcinoma, 47.8%) who had not received any prior treatment and had undergone surgery at Osaka Medical and Pharmaceutical University (OMPU) Hospital. The patients comprised 14 men and 9 women aged between 19 and 76 years (mean age, 57.0 years) who had not received any neoadjuvant therapy. Tumor tissues from patients were fixed in 10% buffered formalin, dehydrated in a graded ethanol series, and embedded in paraffin. Serial sections (3-μm-thick) of the tumor tissues were stained with hematoxylin and eosin (H&E). All histopathological data were reviewed from the corresponding H&E-stained images. PGC was classified according to the pathological tumor-node-metastasis (TNM) classification of the UICC staging system. Patients’ clinicopathological information, including tumor size, stage, nodal metastasis, recurrence, vascular invasion, neuroinvasion, and facial nerve paralysis, was obtained from patients’ medical records and are summarized in Table 1. Vascular invasion was defined as the presence of aggregates of tumor cells within vascular spaces visualized using CD34 (clone QBEnd 10, Dako) staining. Patients’ prognostic information was obtained from patients via a retrospective chart review.

**Table 1:** Correlation between TRKB expression levels and clinicopathological characteristics of patients with PGC (n = 23).

Variables	TRKB		
	Low	High	*P* value
**Gender**			
Male	5	8	0.1333
Female	7	3	
**T classification**			
T1, T2	7	4	0.2921
T3, T4	5	7	
**Stage**			
I, II	7	3	0.1333
III, IV	5	8	
**Nodal metastasis**			
Yes	2	7	0.0211*
No	10	4	
**Recurrence**			
Yes	2	7	0.0211*
No	10	4	
**Vascular invasion**			
Yes	1	9	0.0004***
No	11	2	
**Neuroinvasion**			
Yes	5	8	0.1333
No	7	3	
**Facial nerve paralysis**			
Yes	4	6	0.3053
No	8	5	

Statistical analyses were performed using Pearson’s χ^2^ test or Fisher’s exact test. *P < 0.05; ***P < 0.001.

This study strictly follows the Declaration of Helsinki guidelines for medical research and approved by the Institutional Review Board of OMPU (protocol number: 2382). All experiments were performed in accordance with the guidelines and regulations. Informed consent was obtained from all patients.

### Immunohistochemistry and image analysis

Immunohistochemistry (IHC) of PGC specimens was performed using a BOND-MAX autoimmunostainer (Leica Microsystems) under the same conditions, following a described method in our previous study^14^. Briefly, after removal of endogenous peroxidase activity, paraffin-embedded tissue sections, which contained the deepest site of each tumor, were heated in an antigen unmasking solution and were subsequently incubated with anti-TRKB (1:100; sc-8316, Santa Cruz Biotechnology) and anti-BDNF (1:100; LS-B6557, LifeSpan BioSciences) antibodies. Color development was performed using horseradish peroxidase (HRP)-conjugated secondary antibodies and diaminobenzidine tetrahydrochloride (DAB), and the tissues were counterstained with hematoxylin. Images were obtained using an automated quantitative pathology imaging system (Vectra Polaris, Akoya Biosciences/Kiko Tech). More than five representative areas were captured with high-power microscopic fields (400×) for each PGC tissue specimen. Tissue segmentation into carcinoma and stromal areas was subsequently performed using inForm 2.5 image analysis software. Nuclei were recognized in an automated manner. DAB staining intensity was measured around the nucleus. The positive threshold for staining intensity per cell was defined as > 1% of the maximum staining intensity. Staining intensity was classified into three levels: 1 (low), 2 (low), and 3 (high). The percentage of cells exceeding the threshold was quantified and defined as staining positivity using Spotfire software (TIBICO Software Inc.).

### Establishment and culture of PGC cells and CAFs

PGC cells and CAFs were established from the residual tumor tissues of patients with PGC acquired during surgeries at OMPU Hospital, following a previously described method^25^ with some modifications. Surgical specimens were placed in ice-chilled calcium- and magnesium-free Hanks’ balanced salt solution (HBSS) (Wako Pure Chemical), washed thoroughly with cold HBSS, and cut into cubes of approximately 2 mm^3^.

For isolation of CAFs, tumor fragments were digested with 0.26 U/mL Liberase DH solution (Roche Diagnostics) at 37 °C for 1–2 h. The digested tissues were filtered through 200-μm mesh metal filters and washed several times with cold HBSS. The filtrated cell masses were cultured on culture dishes in RPMI 1640 medium (Gibco, Thermo Fisher Scientific) supplemented with 10% fetal bovine serum (FBS) (Invitrogen), penicillin/streptomycin (100 IU/50 μg/mL), and amphotericin B (0.25 μg/mL) at 37 °C in a humidified atmosphere containing 5% CO_2_. Only spindle-shaped adherent cells that survived after several months of culture and were α-SMA (CAF marker)-positive and VE-cadherin- or PECAM-1 (endothelial cell marker)-negative on immunofluorescence were considered as CAFs.

To establish PGC cells, patient-derived xenograft (PDX) mice were established from residual tumor tissues. Briefly, 2-mm^3^ cubes of tumor fragments were implanted subcutaneously into the flanks of 4- to 5-week-old female NOD.CB17-*Prkdc*^*scid*^/J (NOD SCID) mice (purchased from Charles River Laboratories) until the tumor size reached approximately 1 cm^3^. Surgical specimens from PDX mice were digested and filtered as described above to obtain tumor cell masses. The cell masses were cultured as spheroids on low-attachment culture dishes (PrimeSurface, MS-90350, Sumitomo Bakelite) in StemPro hESC medium (Invitrogen) supplemented with bFGF (8 ng/mL, Wako Pure Chemical), EGF (10 ng/mL, Wako Pure Chemical), penicillin/streptomycin, and amphotericin B^25^. After approximately 1 month of culture, the medium was gradually replaced with RPMI 1640 medium. Once adherent cells appeared and reached 80–90% confluence, they were harvested with 0.05% trypsin–EDTA and subcultured at a 1:4 ratio. The cells were used as PGC cells for the subsequent experiments. All animal work was reviewed and approved by the Osaka Medical College Animal Care & Use committee (protocol number: 21074-A) and follows the ARRIVE guidelines. All experiments were performed in accordance with the guidelines and regulation.

For co-culture assay of PGC cells with CAFs, CAFs were subcultured at 1:2 to 1:3 split ratio in culture dishes. After reaching subconfluence (2–3 days), PGC cells were additionally seeded on the culture dish (1.2 × 10^6^ cells/10-cm dish or 0.2 × 10^6^ cells/35-mm dish) and co-cultured with CAFs for another 4 days. For preparation of cell-free CM, the supernatant of the CM was centrifuged and filtered (0.22 μm) to remove some debris.

### Immunofluorescence

PGC cells and CAFs were cultured for 72–96 h on 0.1% gelatin-coated glass coverslips, fixed in 4% paraformaldehyde (PFA) in phosphate-buffered saline (PBS) for 15–30 min, permeabilized for 5 min with 0.1% Triton X-100 in PBS, and blocked with 3% bovine serum albumin (BSA) (Nacalai Tesque) in PBS for 1 h at room temperature (22–25 °C). The cells were then stained with primary antibodies against TRKB (1:200), BDNF (1:200; sc-546, Santa Cruz Biotechnology), E-cadherin (1:200; #3195, Cell Signaling Technology), N-cadherin (1:250; #14215, Cell Signaling Technology), PECAM-1 (1:1,000; clone MEC13.3, BD Pharmingen), Vimentin (1:250; #5741, Cell Signaling Technology), α-smooth muscle actin (α-SMA) (1:1,000; clone 1A4, Sigma-Aldrich), and Snail (1:250; #3879, Cell Signaling Technology) for 3 h at room temperature. Samples were then incubated with Alexa Fluor 488-conjugated donkey anti-rabbit IgG (Invitrogen) or Cy3-conjugated donkey anti-mouse IgG (Jackson Laboratory) secondary antibodies (1:500 dilution) for 1 h at room temperature. Isotype-matched rabbit IgG (Chemicom International) and mouse IgG (Upstate Biotechnology) antibodies were used for control staining. Alexa Fluor 633-conjugated phalloidin (1:5000; Invitrogen) and DAPI (0.1 μg/mL; 4',6-diamidino-2-phenylindole; Invitrogen) counterstaining was performed. The stained specimens were examined using a fluorescence microscope (BZ-X710, Keyence). Snail- or BDNF-positive cells per field (3500 μm^2^) were counted using the threshold images (Image J) and percentages of Snail- or BDNF-positive cells were calculated.

### Western blot analysis

PGC cells and CAFs were cultured with BDNF or cell-free conditioned medium (CM) in the presence or absence of TRK inhibitors and were lysed in SDS-PAGE sample buffer. Lysates containing equal amounts of protein were used as samples for SDS-PAGE, and Western blotting was performed using PVDF membranes (Merck Millipore) with antibodies against TRKB (1:1000), BDNF (1:1000), E-cadherin (1:1000), N-cadherin (1:1000), VE-cadherin (1:1000; sc-6458, Santa Cruz Biotechnology), α-SMA (1:5000), Vimentin (1:1000), Snail (1:1000), phospho-TRK (1:1000; #4619, Cell Signaling Technology), phospho-Akt (Ser473) (1:1000; #4060, Cell Signaling Technology), phospho-Erk1/2 (Thr202/Tyr204) (1:1000; #9101, Cell Signaling Technology), and β-actin (1:20,000; clone AC-15, Sigma-Aldrich). The membranes were incubated with HRP-conjugated anti-rabbit IgG or anti-mouse IgG antibodies (Jackson Laboratory) and developed using a Luminata Western HRP substrate (Merck Millipore). Signals were detected using the densitometry system Fusion FX7 (Vilber Lourmat).

### Wound healing assay

After reaching subconfluence in 24-well plates, PGC cells were serum-starved in medium containing 0.5% FBS for 24 h, and pre-treated with 100 nM of LOXO-101 (larotrectinib: a pan-TRK inhibitor, 100 nM) or ANA-12 (a TRKB specific inhibitor, 10 μM) for 1 h. Monolayers of confluent PGC cells were subsequently scratched with a sterile 200 μL pipette tip and incubated with BDNF (10 ng/mL) or CM. Phase-contrast images of migrating cells were captured with a 10× objective on an EVOS XL Core Imaging System (Thermo Fisher Scientific). Wound area was measured using ImageJ software. Wound closure percentage was calculated as follows: [(1–wound area at a certain time point)/starting wound area] × 100.

### Statistical analysis

The correlations between TRKB expression and clinicopathological variables were evaluated using Pearson’s χ^2^ test or Fisher’s exact test. The relationships between TRKB expression or tumor subtype and disease-free survival of patients with PGC were evaluated using the Kaplan − Meier method with the log-rank test. Comparisons between two groups were performed using an unpaired two-tailed Student’s *t*-test. Tukey’s honest significant difference test was used for multiple comparisons in the wound healing assay. All statistical analyses were performed using JMP Pro (ver. 15, SAS Institute). Results are presented as the mean ± standard error of the mean (SEM). *P*-values are indicated as follows: ***P < 0.001, **P < 0.01, and *P < 0.05.

## Results

### High expression of BDNF and TRKB in cancer cells and mesenchymal cells in PGC tissues

**Figure 1:**
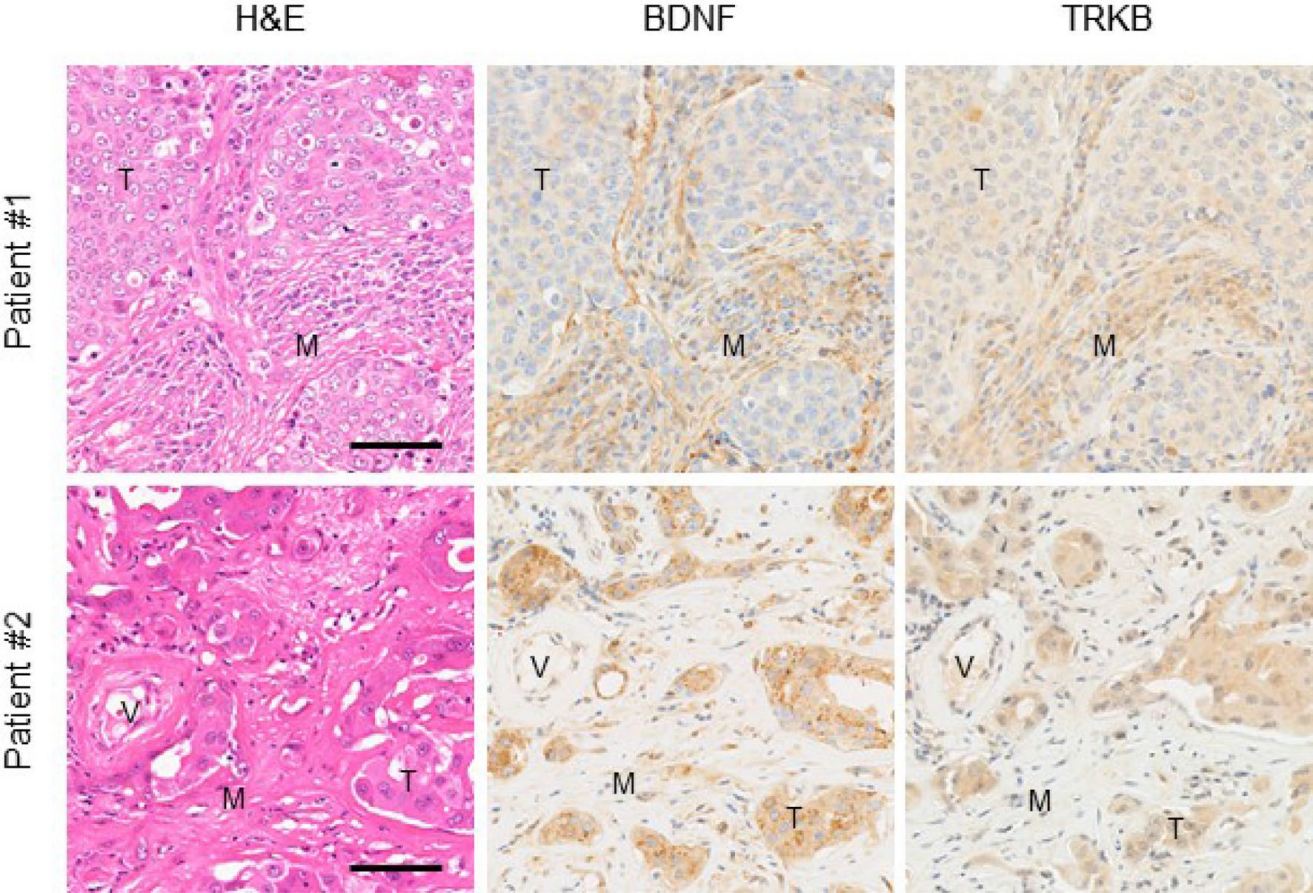
High expression of BDNF and TRKB in cancer cells and mesenchymal cells in PGC tissues. Representative images of H&E and immunostaining of BDNF and TRKB in tumor tissues from patients with PGC. Note that staining for BDNF and TRKB was detected in cancer cells as well as in mesenchymal cells such as fibroblasts and endothelial cells. Scale bars, 100 μm. *T* tumor area, *M* mesenchymal area, *V* vessel.

The tendency of cancer cells to invade neighboring tissues and vasculature is supported by the surrounding niche that secretes multiple growth factors and chemokines. However, the role of the BDNF/TRKB pathway in PGCs is poorly understood. To investigate the involvement of the BDNF/TRKB pathway in the interaction between PGC cells and the surrounding niche, we first examined the distribution and expression levels of BDNF and TRKB in the tumor tissues of patients with PGC using immunohistochemical analysis. As shown in Fig. 1, we identified high expression of BDNF and TRKB in cancer cells as well as in stromal cells such as fibroblasts and vascular endothelial cells. Among the niche cells that populate the tumor microenvironment, CAFs are the most abundant and are critically involved in cancer progression^26, 27^. Notably, involvement of the BDNF/TRKB pathway in the communication between cancer cells and CAFs has been reported for several types of cancer^23, 24^.

### Promotion of EMT and upregulation of BDNF by co-culture with CAFs and inhibition of CAF-mediated upregulation of Snail by a TRKB inhibitor in PGC cells

**Figure 2:**
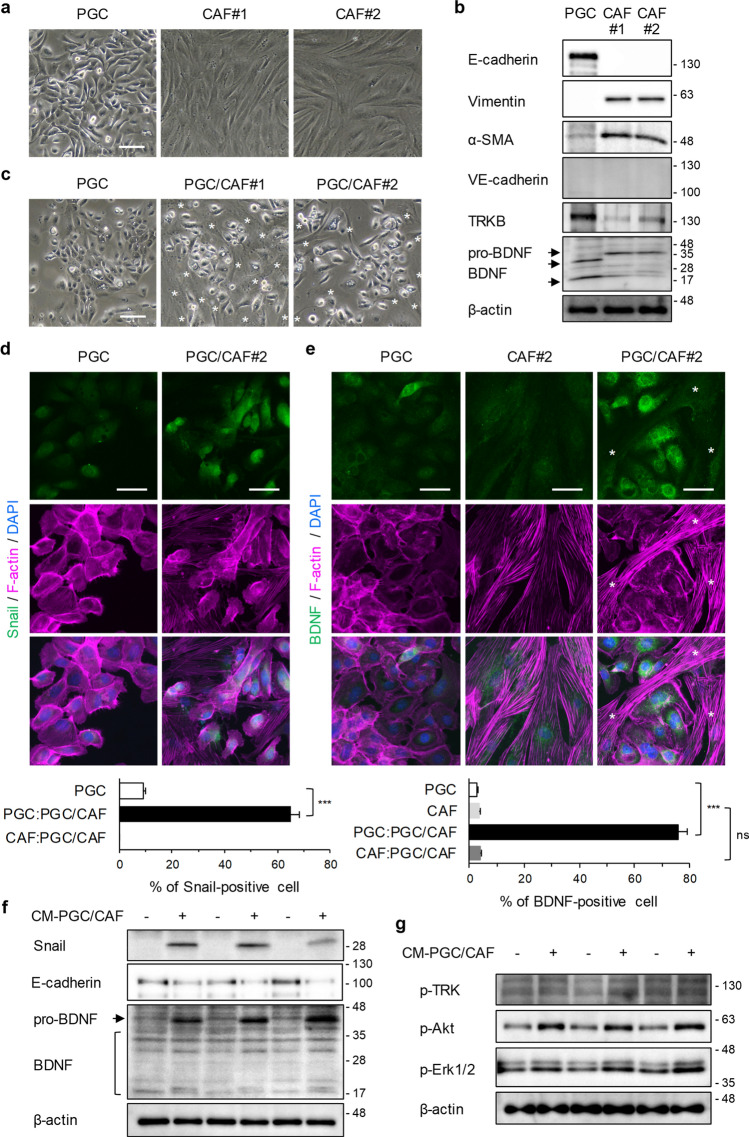
Promotion of EMT and upregulation of BDNF in PGC cells by co-culture with CAFs. **(a)** Representative phase-contrast images of isolated PGCs and CAFs. Scale bar, 200 μm. (**b)** Characterization of established PGC cells and CAFs by WB analysis using the indicated antibodies. (**c–e)** Representative images of phase contrast (**c**) and immunofluorescence staining for Snail (**d**) and BDNF (**e**) in PGC cells which were mono-cultured or co-cultured with CAFs for 4 days. Scale bars, 200 μm (**c**) and 50 μm (**d,e**). Asterisks indicate CAFs. PGC, single-cultured PGC cells; PGC/CAF, direct-co-cultured PGC cells and CAFs. Histograms depict the percentages of Snail- or BDNF-positive cells per field as the mean ± SEM. The percentages were calculated from more than 12 fields (at least 100 cells per field) of each group. PGC:PGC/CAF, direct-co-cultured PGC cells with CAFs; CAF:PGC/CAF, direct-co-cultured CAFs with PGC cells. ***P < 0.001; *ns* not significant (Student’s *t*-test). (**f)** Expression of Snail and BDNF in PGC cells was determined by WB analysis. PGC cells were cultured for 4 days with control cell-free conditioned medium (CM) from PGC cell cultures or CM from PGC/CAF co-cultures. (**g)** Phosphorylation levels of TRK and its down streams, Akt and Erk1/2 were examined by WB analysis. Briefly, PGC cells were serum-starved for 24 h and treated with CM from PGC/CAF co-cultures for 10 min.

To elucidate the molecular mechanisms underlying the role of the BDNF/TRKB pathway in the progression of PGC, we established one PGC cell and two CAF lines from human tumor tissues excised from patients with PGC (see Methods, Fig. 2a). We first confirmed that the cells were PGC cells and CAFs using Western blot and immunofluorescence analyses (Fig. 2b, Supplementary Fig. S1). Isolated PGC cells were mostly positive for E-cadherin (an epithelial marker) and negative for Vimentin, α-SMA, and N-cadherin (mesenchymal markers), whereas isolated CAFs were positive for Vimentin, α-SMA, and N-cadherin and negative for E-cadherin, VE-cadherin, and PECAM-1 (endothelial markers). In addition, TRKB was highly expressed (Fig. 2b) and TRKB phosphorylation was upregulated after BDNF stimulation in cultured PGC cells (Supplementary Fig. S1c). TRKB was moderately expressed in cultured CAFs (Fig. 2b). BDNF was expressed in both PGC cells and CAFs (Fig. 2b). In subsequent studies, we used these established cells as PGC cells and CAFs.

**Figure 3:**
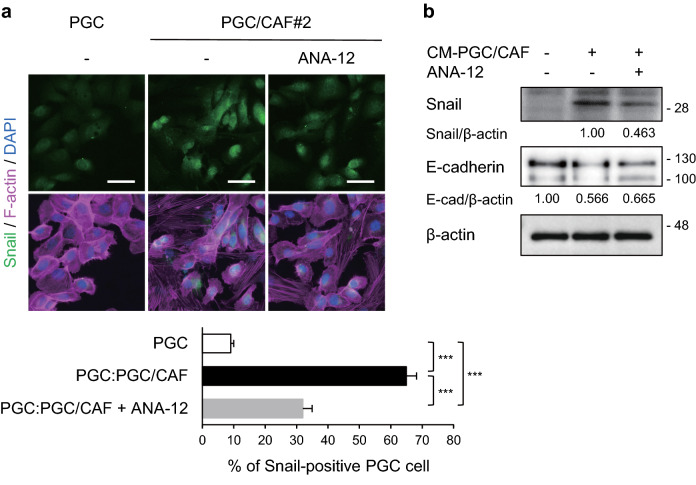
Inhibition of CAF-mediated upregulation of Snail in PGC cells by a TRKB inhibitor. PGC cells were co-cultured with CAFs (**a**) or CM from PGC/CAF co-cultures (**b**) for 4 days in the presence or absence of a TRKB inhibitor, ANA-12 (10 μM). (**a)** Snail expression levels were examined using immunofluorescence analysis. Histogram shows the percentage of Snail-positive cells per field as the mean ± SEM. The percentage were calculated from more than 11 fields (at least 100 cells per field) of each group. PGC:PGC/CAF, direct-co-cultured PGC cells with CAFs. ***P < 0.001 (Tukey’s test). (**b)** Snail and E-cadherin expression levels were examined using WB analysis. The relative amounts of Snail are indicated as the ratio of each protein band relative to the loading control, β-actin. Scale bars, 50 μm.

Since PGC cells co-cultured directly with CAFs exhibited EMT-related features with weakened cell–cell adhesion and elongated cell shape (Fig. 2c), we performed immunofluorescence analysis to confirm whether the changes in molecular expression were caused by EMT. We observed that zinc finger protein SNAI1 (Snail), a master regulatory transcription factor for EMT, was induced in PGC cells co-cultured directly with CAFs (Fig. 2d), indicating that co-cultured PGC cells promoted EMT. Further, BDNF expression was higher in co-cultured PGC cells than in PGC cells cultured alone (Fig. 2e). Snail and BDNF expression levels were also upregulated in PGC cells treated with CM from co-cultures of PGC cells with CAFs but were not altered by treatment with CM from cultures of PGC cells or CAFs (Supplementary Fig. S3, Fig. 2f). In addition, in the PGC cells, E-cadherin expression levels were reduced upon induction of Snail expression. As shown in Supplementary Fig. S2, CM from co-cultures of PGC cells with CAFs included BDNF. WB analysis also show that CM treatment increased phosphorylation levels of TRK and its downstream, Akt and Erk1/2 in PGC cells (Fig. 2g). Immunofluorescence and Western blot analyses revealed that the induction of Snail and reduction of E-cadherin in PGC cells co-cultured directly with CAFs or treated with co-cultured CM were partially inhibited (approximately 54% and 22% inhibition) by a TRKB inhibitor, ANA-12 (Fig. 3). These data suggest that the crosstalk between PGC cells and CAFs promotes EMT at least partly via activation of the BDNF/TRKB pathway in PGC cells by soluble factors produced by co-culture with CAFs.

### Suppression of BDNF-induced cell migration of PGC cells by TRK inhibitors

**Figure 4:**
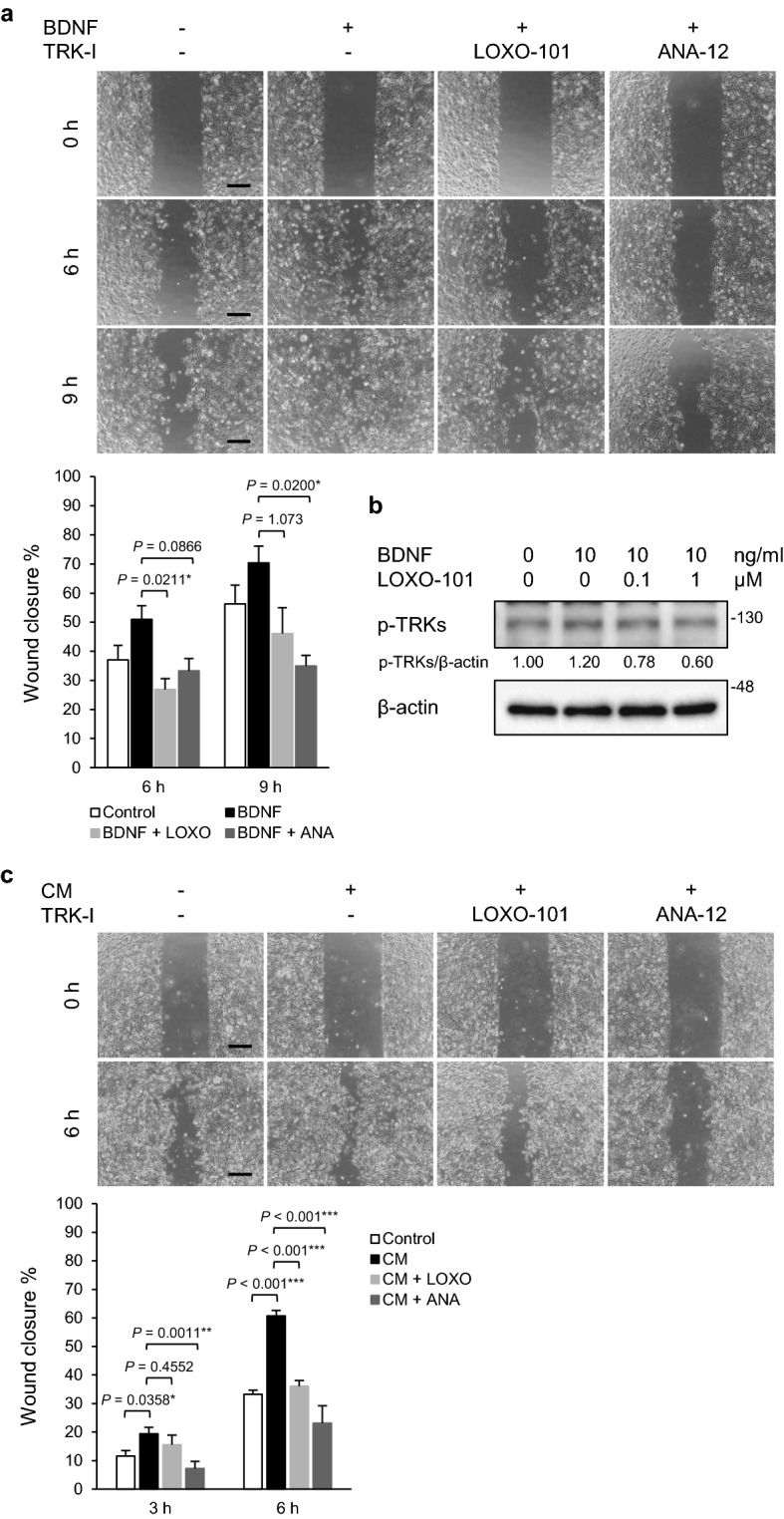
Suppression of BDNF-induced cell migration of PGC cells by TRK inhibitors. **(a)** Wound healing assay. After serum starvation (0.5% FBS for 24 h), PGC cells were pre-treated with LOXO-101 (a pan-TRK inhibitor, 100 nM) or ANA-12 (a TRKB specific inhibitor, 10 μM) for 1 h, and a wound healing assay was performed. The migrating cells were imaged and cell migration was estimated as wound closure percentage at the indicated time points (0, 6, and 9 h). Histograms depict the percentages of wound closure as the means ± SEM of triplicate wells from three independent experiments. Scale bars, 100 μm. *P < 0.05 (Tukey’s test). (**b)** Phosphorylation of TRKB in PGC cells was determined by WB analysis. After serum starvation (0.5% FBS for 24 h), PGC cells were pre-treated with LOXO-101 (100 nM) for 1 h and subsequently cultured in the presence or absence of BDNF (10 ng/mL) for 30 min. The relative amounts of p-TRKs are indicated as the ratio of each protein band relative to the loading control, β-actin. (**c)** Wound healing assay with CM from PGC/CAF co-cultures. Data shown are means ± SEM of quintuple wells and are representative of three independent experiments. TRK-I, TRK inhibitor. ***P < 0.001, **P < 0.01, and *P < 0.05 (Tukey’s test).

The BDNF/TRKB pathway mediates tumor cell migration and invasion^9, 14, 16, 17^. As described above, BDNF was secreted from PGCs and mesenchymal cells such as CAFs (Fig. 1), and treatment with a TRKB inhibitor partially blocked the upregulation of Snail in PGC cells via interaction with CAFs (Fig. 3), indicating that the BDNF/TRKB pathway may promote PGC cell motility. To verify this, BDNF-induced cell migration of PGC cells was examined using a wound healing assay (Fig. 4a). PGC cell migration tended to be upregulated by BDNF stimulation, and this upregulation was completely inhibited by treatment with LOXO-101 (a pan-TRK inhibitor) or ANA-12 (a TRKB-specific inhibitor). These results were consistent with the finding that TRKB phosphorylation was increased by BDNF treatment, and this increase was attenuated by LOXO-101 treatment in a dose-dependent manner (Fig. 4b). Additionally, CM from co-cultures of PGC cells with CAFs also induced cell migration of PGC cells and the migration was significantly inhibited by treatment with LOXO-101 or ANA-12 (Fig. 4c).

### High TRKB expression levels in PGC cells and association with poor prognosis in patients with PGC

**Figure 5:**
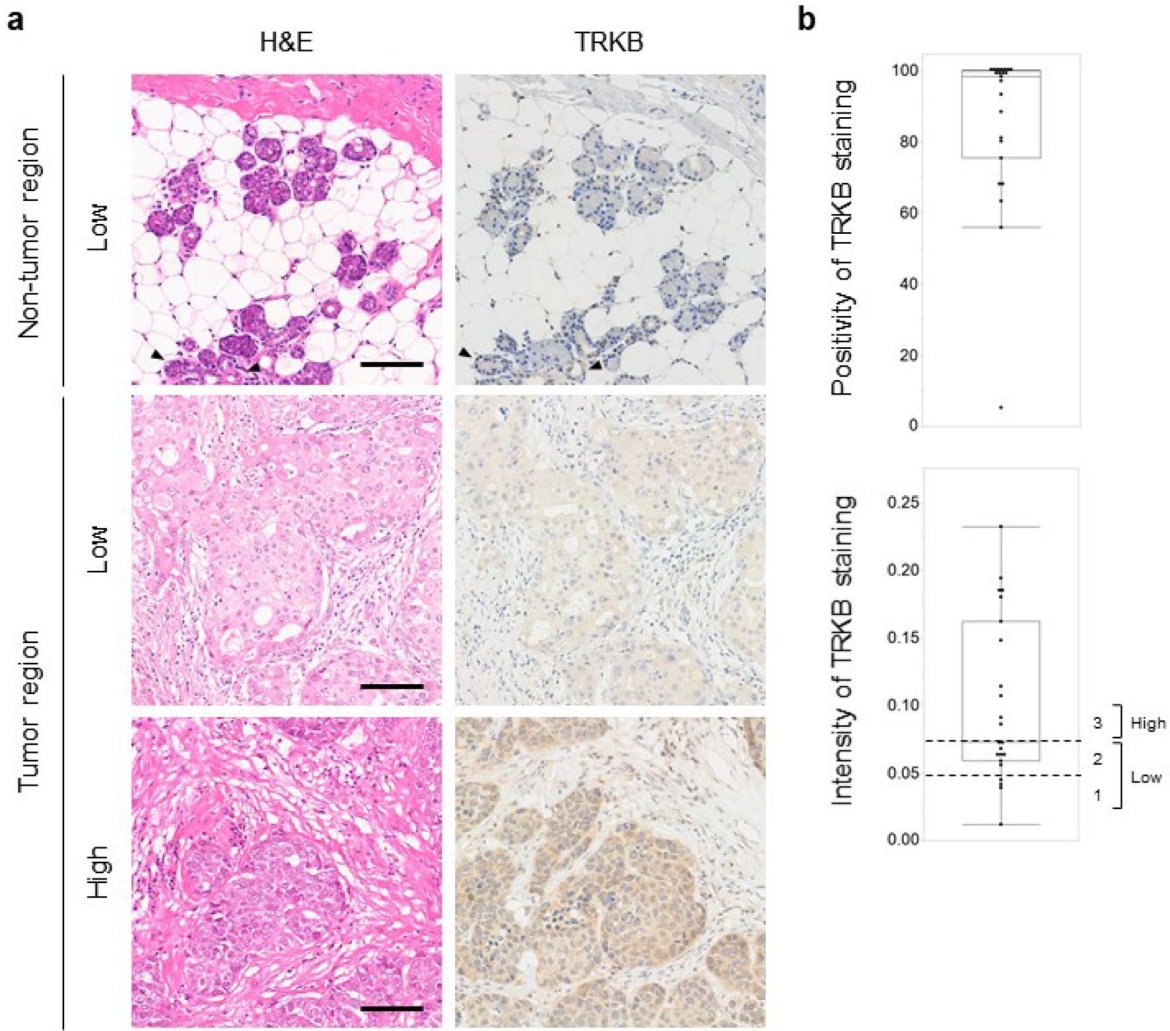
High expression of TRKB in PGCs. **(a)** Representative H&E and TRKB staining images of tumor tissues from patients with PGC. Arrowheads indicate ductal epithelial cells with faint expression of TRKB. Scale bars, 100 μm. (**b)** TRKB expression levels in each type of PGC. Positivity and intensity of TRKB staining in cancer cells were analyzed using inForm and Spotfire software. TRKB expression levels were classified into three levels (staining intensity: < 0.05, level 1; 0.05–0.75, level 2; > 0.75, level 3) and divided into two groups: low (levels 1 and 2) and high (level 3).

Our primary cell culture system demonstrated that TRKB expression in PGC cells contributed to the promotion of malignant behavior in PGC cells, suggesting that TRKB expression may be a key prognostic marker in patients with PGC. To clarify the clinical significance of TRKB expression in patients with PGC, we first analyzed the expression levels of TRKB in specimens excised from 23 patients with PGC that had not undergone presurgical chemotherapy. Immunohistochemical staining for TRKB revealed high TRKB expression in the tumor regions of PGC tissues (Fig. 5a). TRKB was undetectable in normal-appearing tissues adjacent to tumor tissues, except for the ductal epithelium, which exhibited faint expression. To evaluate TRKB expression levels, the signal intensity of TRKB staining was measured using inForm 2.5 image analysis software (Supplementary Fig. S4), and TRKB staining positivity and intensity were calculated (Fig. 5b). Most PGCs exhibited more than 50% TRKB-positive tumor cells and diverse TRKB expression levels. Accordingly, we divided the samples into two groups, i.e., low TRKB (TRKB^low^) and high TRKB (TRKB^high^) (see Methods, Fig. 5b), and analyzed the correlation between TRKB expression levels and the clinical significance of PGC.

Table 1 presents the relationships between TRKB expression levels and clinicopathological features of patients with PGC. No significant correlations were observed between TRKB expression and sex, T classification, clinical stage, neuroinvasion, and facial nerve paralysis. In contrast, TRKB expression was significantly correlated with nodal metastasis (*P* = 0.0211), recurrence (*P* = 0.0211), and vascular invasion (*P* = 0.0004). The number of patients with the poorest prognostic markers increased in a stepwise manner depending on TRKB expression levels classified into three levels (levels 1, 2, and 3) (Supplementary Fig. S5).

**Figure 6:**
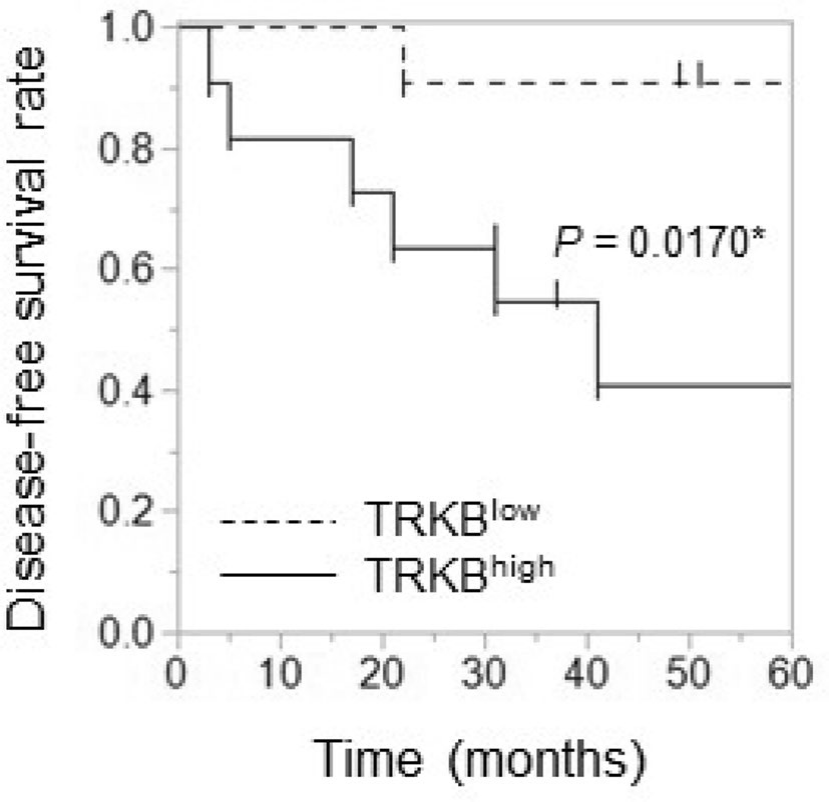
Poor prognosis in patients with PGC with high TRKB expression. Relationships between 5-year disease-free survival and expression levels of TRKB were analyzed using the Kaplan − Meier method. *P < 0.05.

To clarify the association between TRKB expression levels and PGC prognosis, the 5-year survival rate was analyzed using the Kaplan − Meier method. As shown in Fig. 6, disease-free survival rate was significantly lower in patients with TRKB^high^ than in those with TRKB^low^ (*P* = 0.0170). These findings suggest that TRKB^high^ represents a risk factor for recurrence and unfavorable prognosis in patients with PGC.

Collectively, these data imply that TRKB expression levels are significantly elevated in PGC and that high TRKB expression is associated with vascular invasion, nodal metastasis, and poor prognosis.

## Discussion

Cancer classification based on protein expression using clinicopathological and immunohistochemical analyses of human clinical specimens is valuable for individual patients with cancer for clinical decision-making and the development of unknown clinical potential, i.e., potential biomarkers for disease prognosis. As the BDNF/TRKB pathway has been reported to promote metastasis in several types of cancers^9, 11, 19^, we focused on BDNF/TRKB pathway-related proteins in cancer tissues from patients with PGC. We observed that BDNF and TRKB were highly expressed in both cancer cells and mesenchymal cells (Fig. 1). To examine the involvement of the BDNF/TRKB pathway in the interaction between PGC cells and the surrounding niche, we established PGC cells and CAFs from patients with PGC. Our primary co-culture system of PGC cells and CAFs demonstrated that BDNF and Snail expression was increased in PGC cells by direct or indirect interaction with CAFs (Fig. 2, Supplementary Fig. S3), indicating the involvement of crosstalk in BDNF/TRKB pathway activation and promotion of EMT. CAF-mediated upregulation of Snail and BDNF-induced cell migration in PGC cells was attenuated by treatment with a TRKB-specific inhibitor or an anticancer drug, larotrectinib (a pan-TRK inhibitor) (Figs. 3 and 4). Furthermore, we determined the molecular features associated with clinicopathological variables and their significance in cancer progression using clinical tumor samples from patients with PGC. High TRKB expression was significantly correlated with aggressive features such as vascular invasion, nodal metastasis, and poor prognosis (Table 1, Fig. 6). These data indicate that the BDNF/TRKB pathway is involved in the aggressiveness of PGC and may be an appropriate therapeutic target for aggressive PGC with poor prognosis.

Overexpression of TRK proteins is predominantly induced by *NTRK* gene copy number gain rather than *NTRK* gene fusions^28, 29^. Moreover, hypoxia induces *NTRK2* gene expression in tumor cells via HIF-1α and upregulates BDNF and TRKB expression in tumor cells, which can aggravate and prolong the effects of the BDNF/TRKB pathway in tumors^30^. However, little is known about TRK overexpression in tumor cells that are regulated by communication with the tumor microenvironment. The contribution of CAFs, the most abundant cell population in the tumor niche, to tumor progression by gene expression changes in tumor cells, has been extensively reported. A previous study reported that a normal fibroblast cell line produced BDNF in co-cultures with an oral tumor cell line and subsequently induced upregulation of TRKB expression and EMT in tumor cells^23^, in support of CAF-mediated activation of the BDNF/TRKB pathway. Here, we demonstrated that CAFs substantially upregulated BDNF expression in PGC cells in our primary co-culture system of PGC cells with CAFs. Although CM from co-cultures of PGC cells with CAFs did not increase BDNF expression in CAFs (Fig. 2e), our data do not exclude the possibility that BDNF, which is secreted by CAFs, regulates cancer cell functions. This is because high BDNF expression was observed in PGC cells as well as in stromal cells such as CAFs in immunohistochemical analyses of human clinical PGC specimens (Fig. 1). These findings indicate that the BDNF/TRKB pathway may interact with cancer cells and CAFs in a paracrine manner. A previous report demonstrated that vascular endothelial cell-derived BDNF promotes cancer cell migratory ability^31^. We observed BDNF expression in stromal cells, including endothelial cells and CAFs. We previously reported that high TRKB expression in head and neck squamous cell carcinoma (HNSCC) is correlated with vascular invasion and disease-free survival in patients with HNSCC and that tumor cells and tumor-associated vessels in clinical HNSCC tumors exhibit aberrant BDNF and TRKB expression^14^. Consistent with these findings, a similar correlation was observed in patients with PGC (Table 1, Fig. 6), and the BDNF and TRKB expression in vessels was also observed in PGC tissues (Fig. 1). These findings indicate that the BDNF/TRKB pathway functions in the crosstalk between cancer cells and the tumor niche, including CAFs and endothelial cells via both autocrine and paracrine mechanisms in multiple processes of tumor progression, including tumor cell invasion and metastasis. In in vitro PGC culture systems, BDNF stimulation promoted cell migration in PGC cells. Cell migration was suppressed to the levels of control (non-treated) groups by treatment with ANA-12 (a TRKB-specific inhibitor) or larotrectinib (a pan-TRK inhibitor) (Fig. 4a). These data suggest that BDNF functions in an autocrine manner in PGC cells. The fact that larotrectinib effectively inhibits BDNF suggests that other TRK family members may also contribute to PGC cell migration.

In conclusion, we demonstrated that the BDNF/TRKB pathway was upregulated in PGC cells by crosstalk with CAFs, and BDNF-induced migration in PGC cells was suppressed by TRK inhibitors. Further, we observed high TRKB expression in clinical PGC that was correlated with poor prognosis, including vascular invasion, nodal metastasis, recurrence, and survival rate in patients with PGC. These results suggest that the BDNF/TRKB pathway may promote PGC cell aggressiveness via crosstalk with CAFs. Our findings highlight the BDNF/TRKB axis as a potential therapeutic target for PGC harboring invasive and metastatic features.

## Supplementary Information

Supplementary Figures.

## Data Availability

All data generated or analysed during this study are included in this published article (and its Supplementary Information files).

## Abbreviations

PGC: Parotid gland cancer
OSCC: Oral squamous cell carcinoma
TNM: Tumor-node-metastasis
PDX: Patient-derived xenograft
TRK: Tropomyosin receptor kinase
BDNF: Brain-derived neurotrophic factor
PGC cells: Patient-derived PGC cells
CAFs: Cancer-associated fibroblasts
EMT: Epithelial-mesenchymal transition
PBS: Phosphate-buffered saline
BSA: Bovine serum albumin
FBS: Fetal bovine serum
DAPI: 4',6-Diamidino-2-phenylindole
CM: Cell-free conditioned medium
TBS: Tris-buffered saline
HBSS: Hanks’ balanced salt solution
DMSO: Dimethyl sulfoxide
IHC: Immunohistochemistry
H&E: Hematoxylin and eosin
HRP: Horseradish peroxidase
DAB: Diaminobenzidine tetrahydrochloride
PFA: Paraformaldehyde
