# Supplementary material for: BDNF/TRKB axis provokes EMT progression to induce cell aggressiveness via crosstalk with cancer-associated fibroblasts in human parotid gland cancer
Source: Sci Rep. 2022 Oct 20;12:17553. doi: 10.1038/s41598-022-22377-9 (PMC9584965; doi:10.1038/s41598-022-22377-9)

Supplemental Data

BDNF/TRKB axis provokes EMT progression to induce cell aggressiveness via crosstalk with cancer-associated fibroblasts in human parotid gland cancer

Kazumasa Moriwaki[1,*,+], Masaki Wada[2,+], Hiroko Kuwabara[3], Yusuke Ayani[2], Tetsuya Terada[2], Masaaki Higashino[2], Ryo Kawata[2], and Michio Asahi[1,*]

[1]Department of Pharmacology, Faculty of Medicine, Osaka Medical and Pharmaceutical University, 2-7, Daigaku-machi, Takatsuki, Osaka 569-8686, Japan
[2]Department of Otolaryngology-Head and Neck Surgery, Faculty of Medicine, Osaka Medical and Pharmaceutical University, 2-7, Daigaku-machi, Takatsuki, Osaka 569-8686, Japan
[3]Department of Pathology, Faculty of Medicine, Osaka Medical and Pharmaceutical University, 2-7, Daigaku-machi, Takatsuki, Osaka 569-8686, Japan
*Authors for correspondence: E-MAIL masahi@ompu.ac.jp or kazumasa.moriwaki@ompu.ac.jp; Phone 81-72-684-7292; FAX 81-72-684-6518
[+]These authors contributed equally to this work.

Supplementary Figure S1

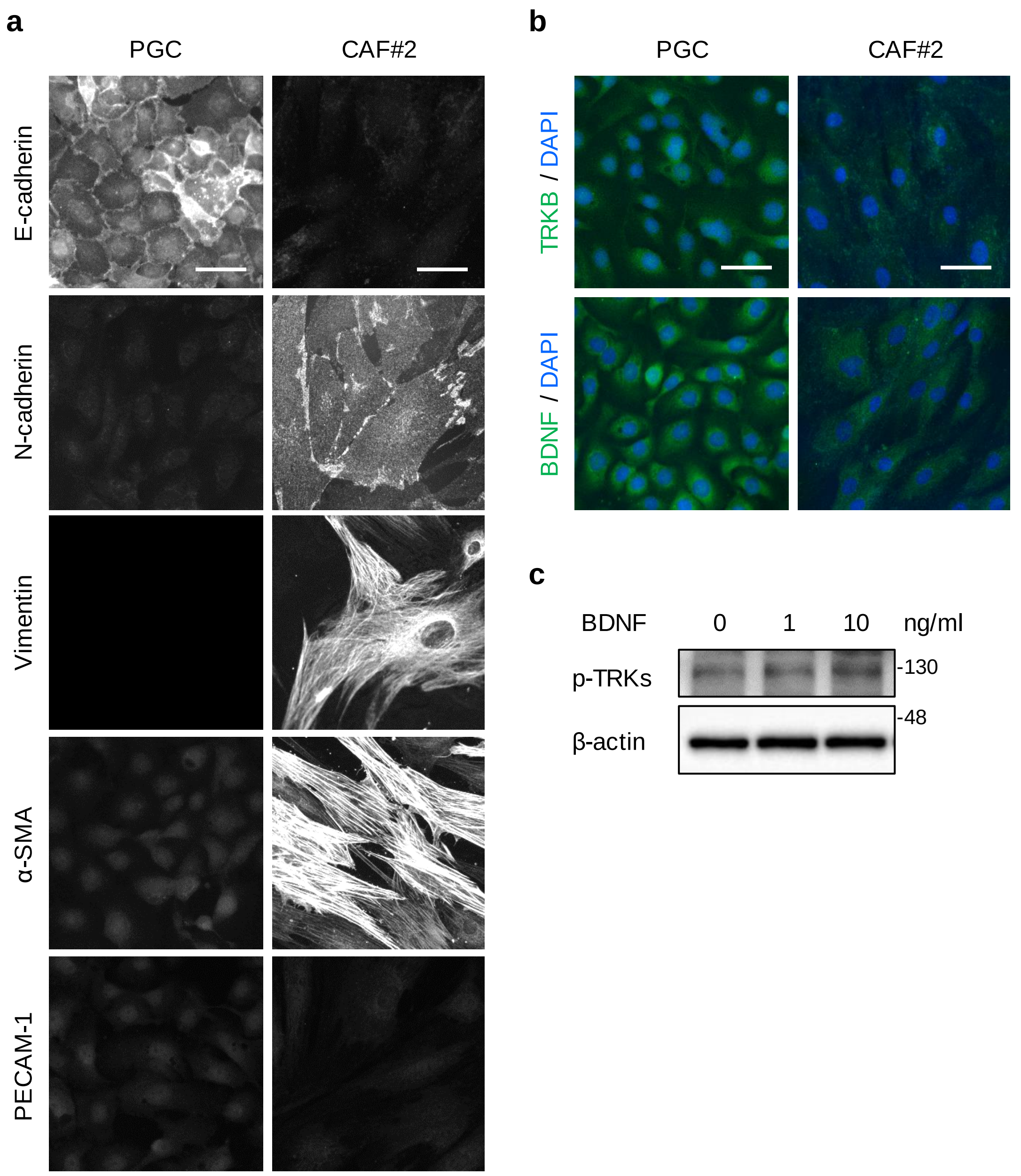

Supplementary Figure S1. Establishment of PGC cells and CAFs from patients with PGC.

a. Characterization of established PGC cells and CAFs by immunofluorescence using the indicated antibodies. Scale bars, 50 µm. **b.** Expression of TRKB and BDNF in PGC cells and CAFs was detected by immunofluorescence analysis. Scale bars, 50 µm. **c.** BDNF-dependent phosphorylation of TRKB in PGC cells was determined by WB analysis using anti-phospho-TRK antibody (1:1000; #4619, Cell Signaling Technology). After 24 h of serum-starvation (0.5% FBS), PGC cells were stimulated with BDNF (0, 1, and 10 ng/ml) for 30 min.

Supplementary Figure S2

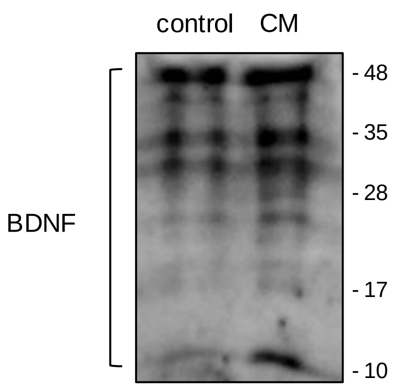

Supplementary Figure S2. BDNF production in conditioned medium.

BDNF in CM from PGC/CAF co-culture was determined by WB analysis using anti-BDNF antibody. Basal medium (RPMI1640 with 10% FBS) cultured for 4 days in a cell culture incubator was used as control.

Supplementary Figure S3

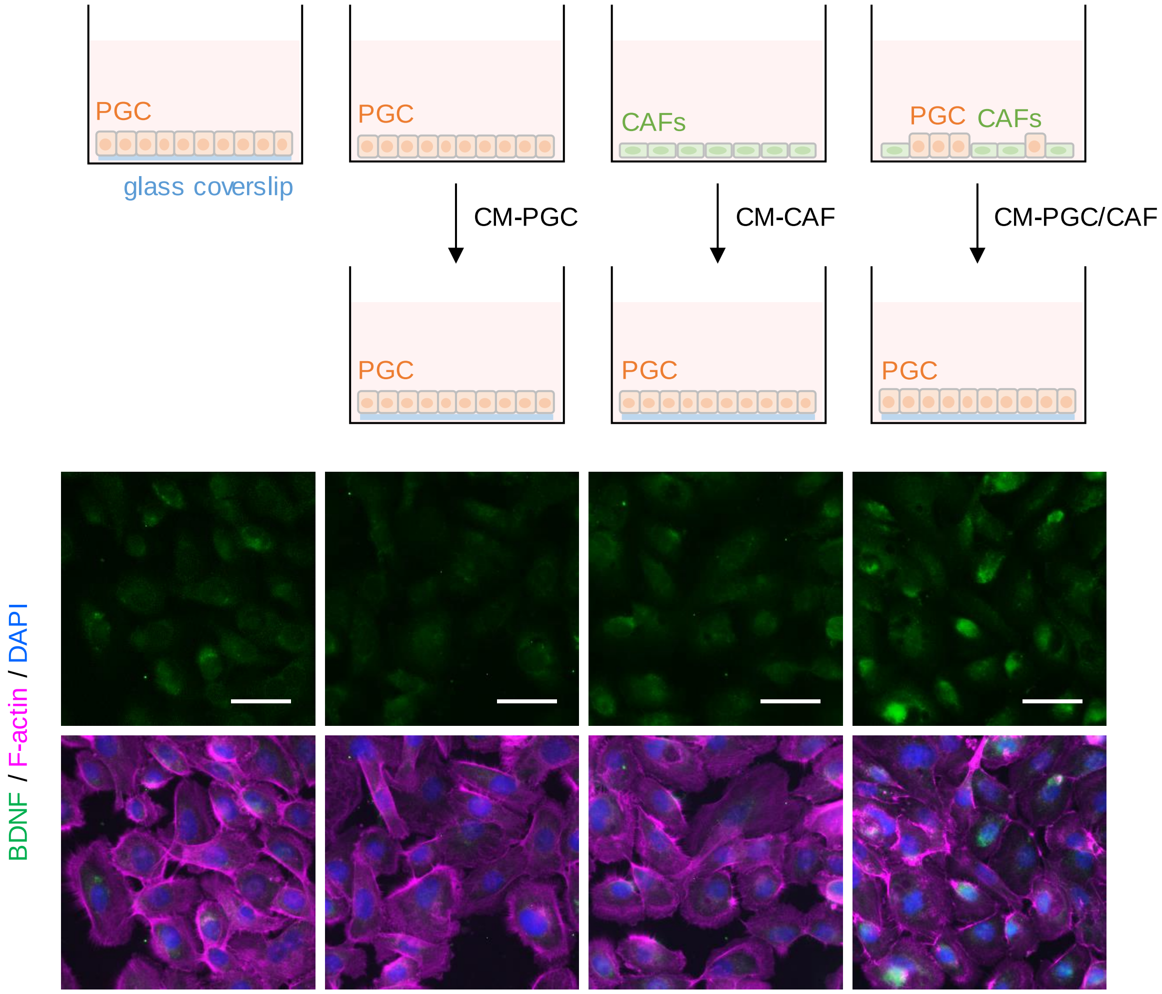

Supplementary Figure S3. Upregulation of BDNF in PGC cells directly or indirectly co-cultured with CAFs. BDNF expression in PGC cells was determined by immunofluorescence analysis. PGC cells were cultured for 4 days under the conditions described in the scheme. Scale bars, 50 μm. CM-PGC, cell-free conditioned medium from PGC cultures; CM-CAF, cell-free conditioned medium from CAF cultures; CM-PGC/CAF, cell-free conditioned medium from PGC/CAF co-cultures.

Supplementary Figure S4

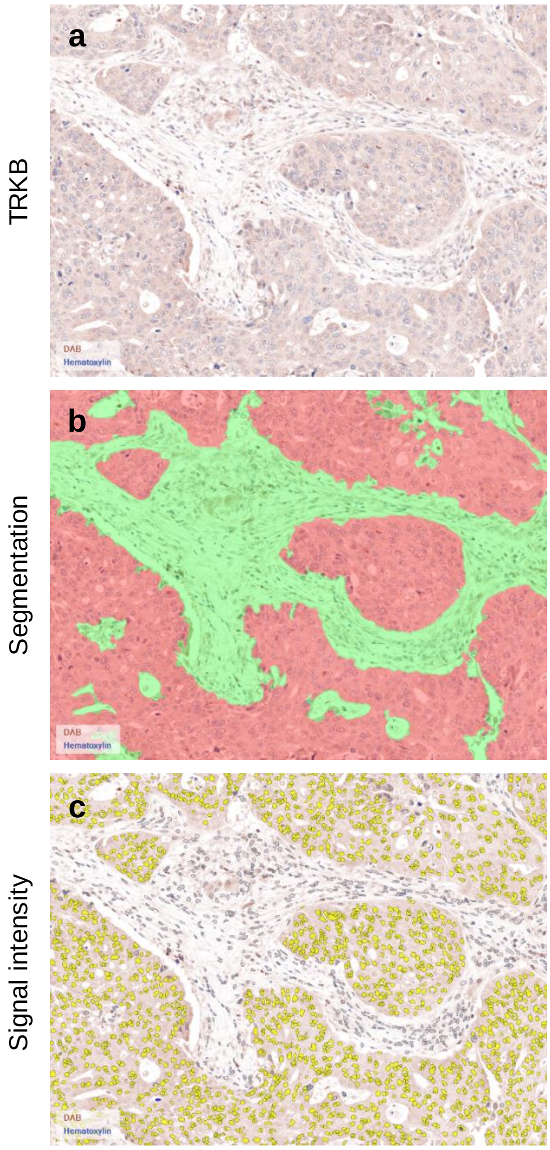

Supplementary Figure S4. Imaging analysis of immunohistochemistry by inForm software.

a. Representative image of TRKB-staining tissue acquired using Vectra Polaris automated quantitative pathology imaging system. **b-c.** Tissue segmentation into carcinoma (red) and stromal areas (green) and the staining positivity (blue, negative; yellow, positive) were shown in (b) and (c), respectively.

Supplementary Figure S5

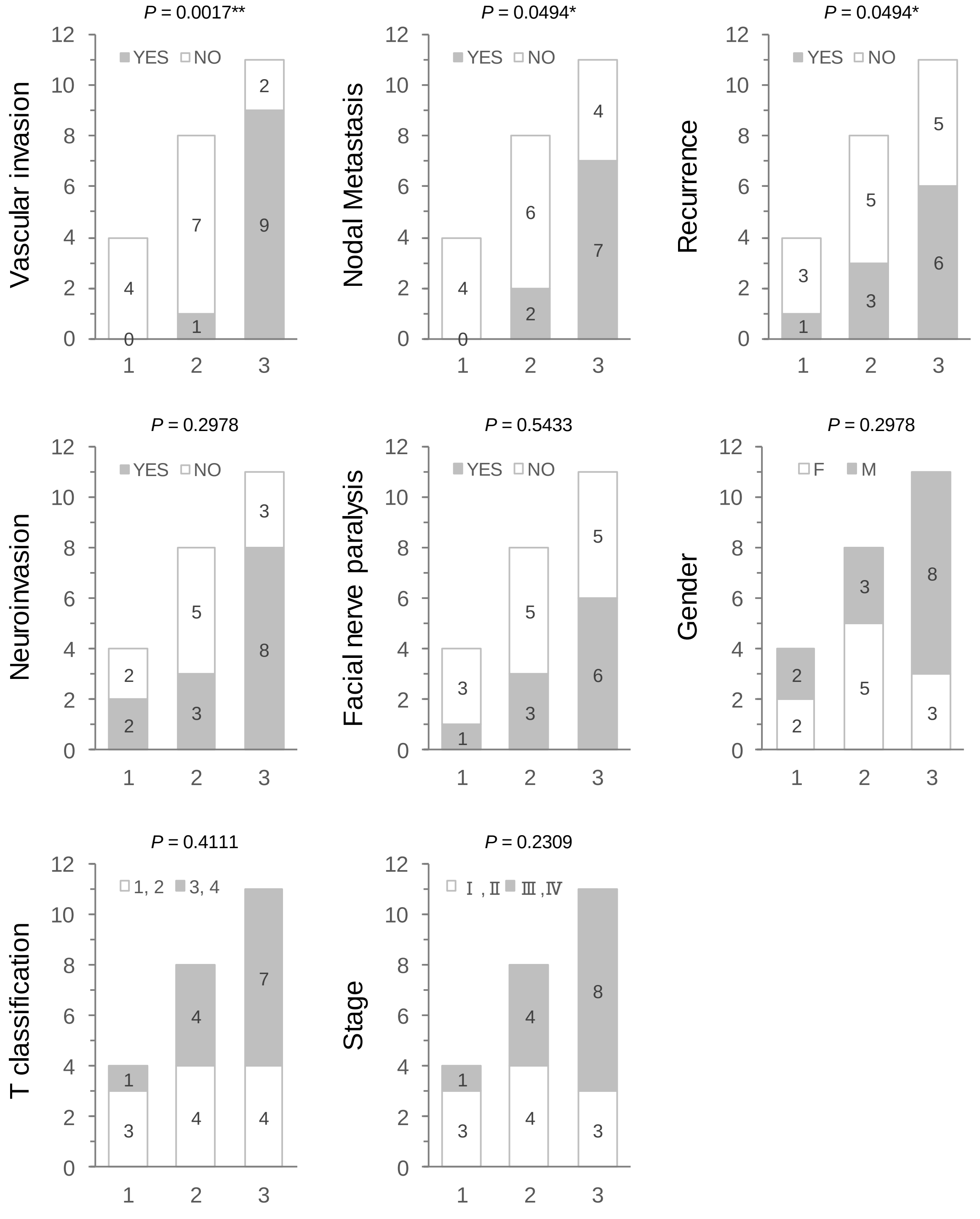

Supplementary Figure S5. Correlation between TRKB expression and clinical significance of patients with PGC (n = 23).

Histograms show the correlation between the TRKB-staining intensity which was classified into three levels (level 1 < 2 < 3, see Fig. 5b) and the clinical variables. Note, the number of patients with the poorest prognostic markers increased in a stepwise manner depending on TRKB expression levels. F, female; M, male. Statistical analyses were performed using Pearson's χ^2 test or Fisher's exact test. **P* < 0.05; ***P* < 0.01.

Supplementary Figure S6. Original images of Western blot.

Original images of Western blot or PVDF membrane in all experiments are shown below.

Fig. 2b. E-cadherin
Vimentin

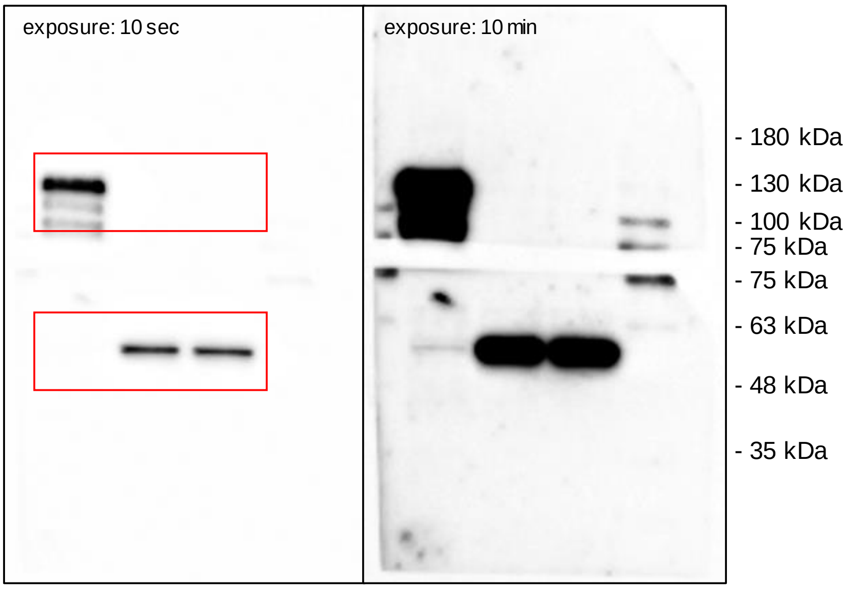

Fig. 2b. α-SMA

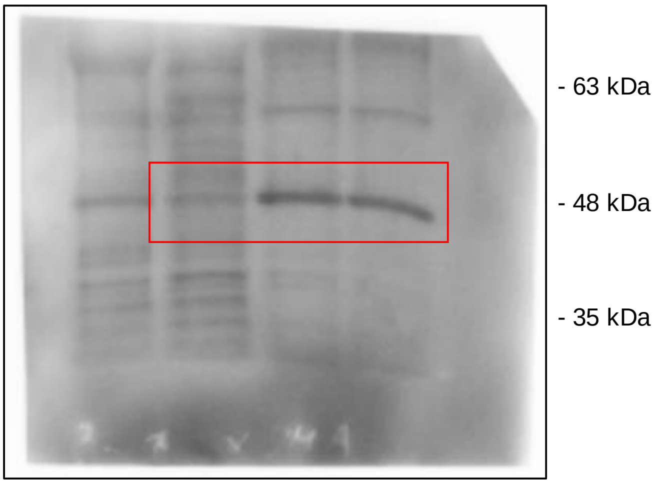

Fig. 2b. VE-cad

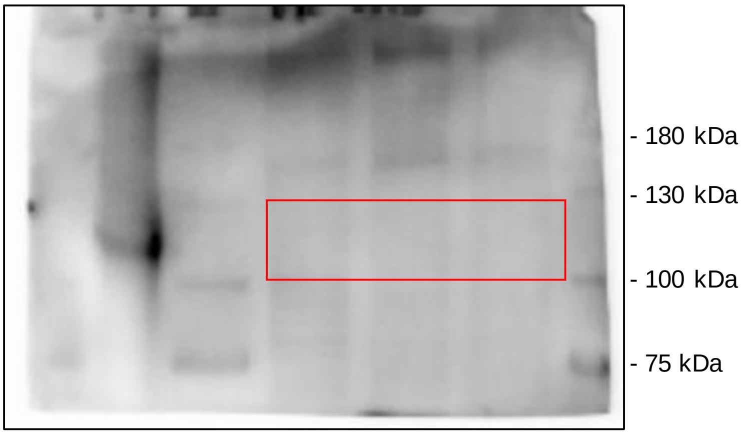

Fig. 2b. TRKB

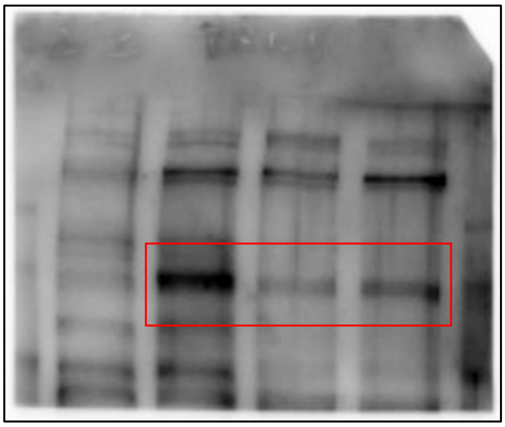

Fig. 2b. BDNF

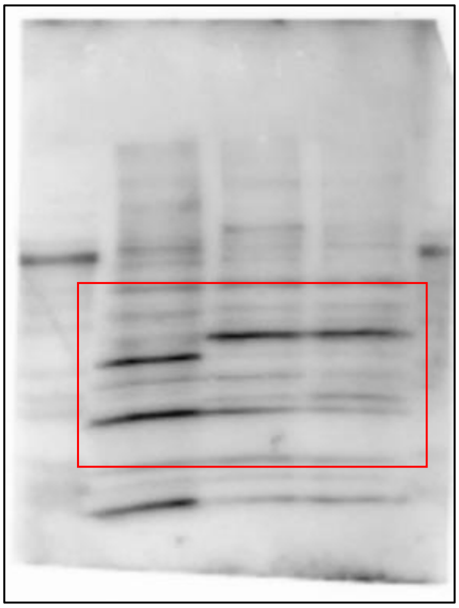

Fig. 2b. β-actin

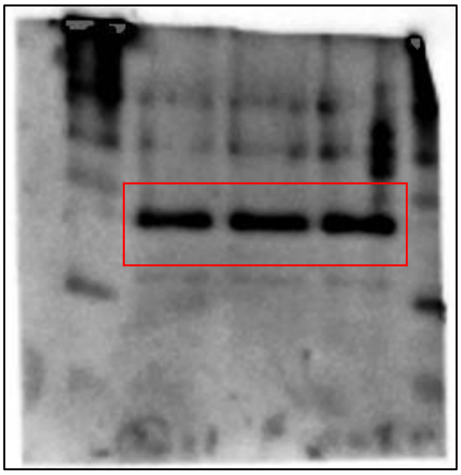

Fig. 2f. Snail

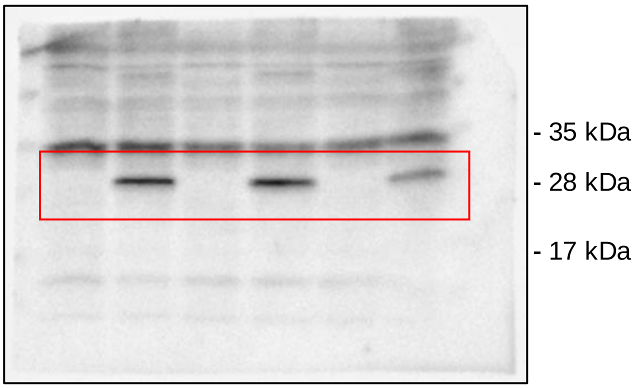

Fig. 2f. E-cadherin

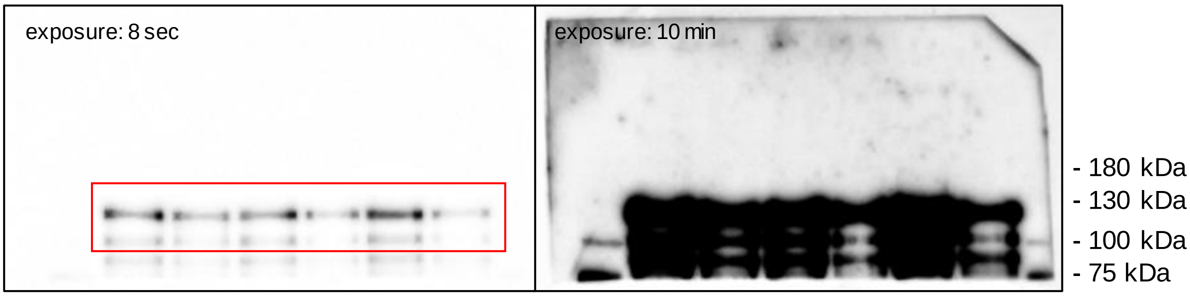

Fig. 2f. BDNF

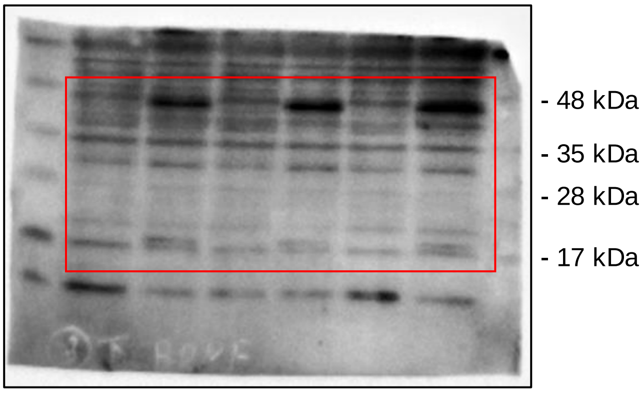

Fig. 2f. β-actin

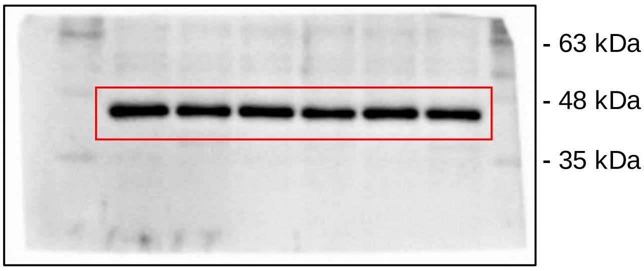

Fig. 2g. p-TRK

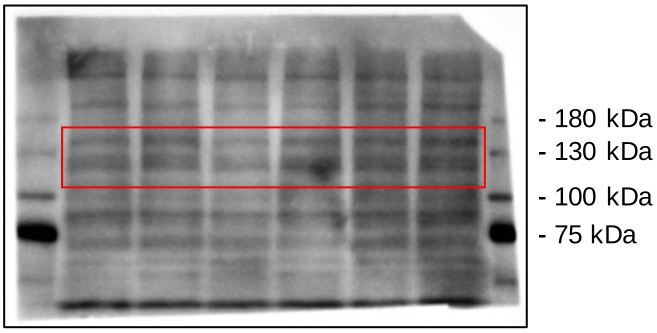

Fig. 2g. p-Akt

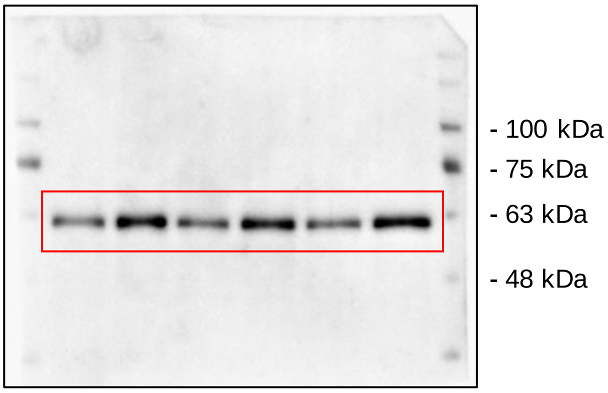

Fig. 2g. p-Erk1/2

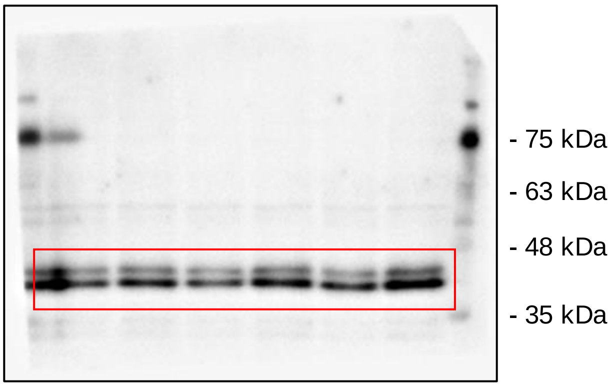

Fig. 2g. β-actin

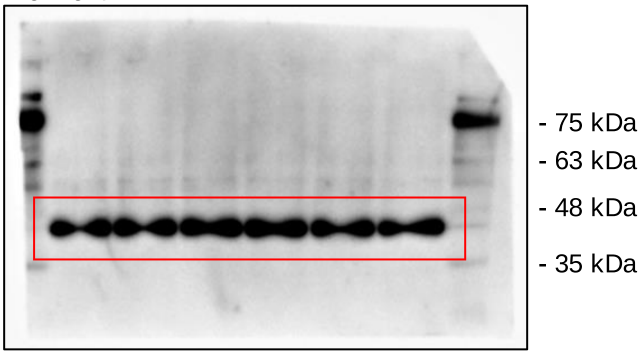

Fig. 3b. Snail

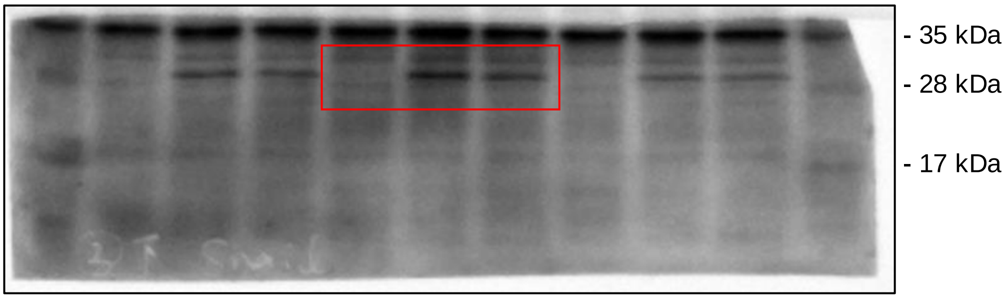

Fig. 3b. E-cadherin
β-actin

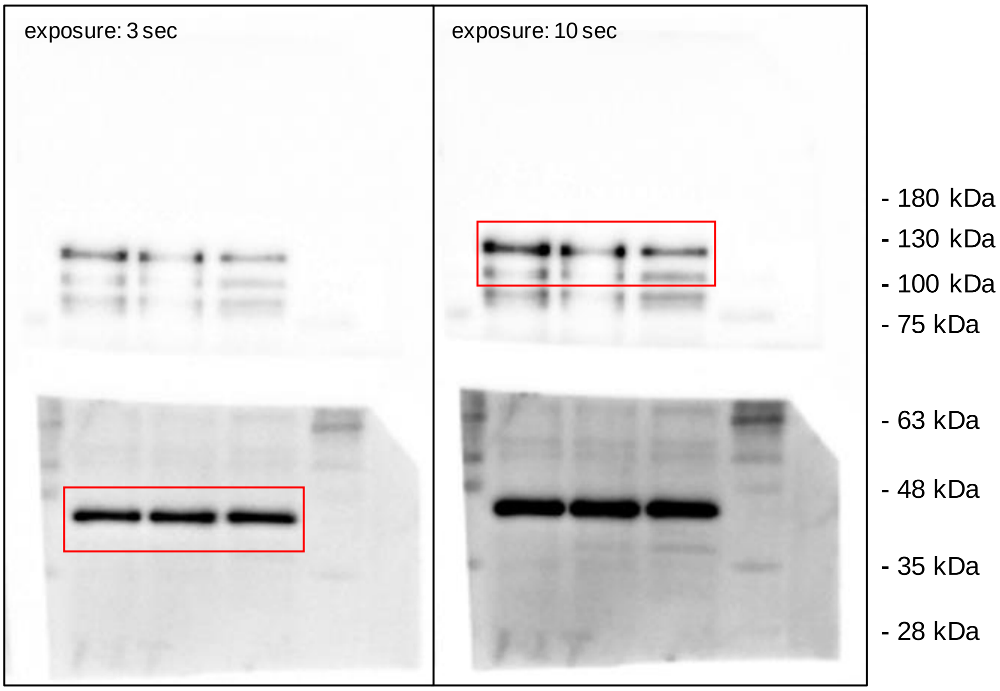

Fig. 4b. p-TRKs

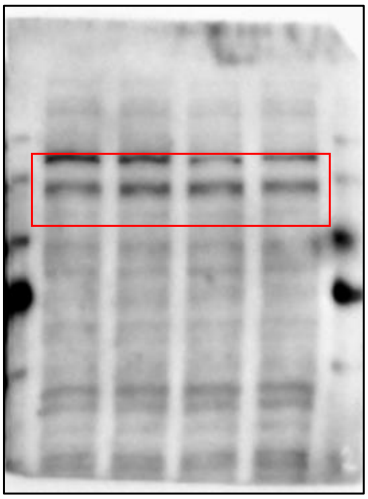

Fig. 4b. β-actin

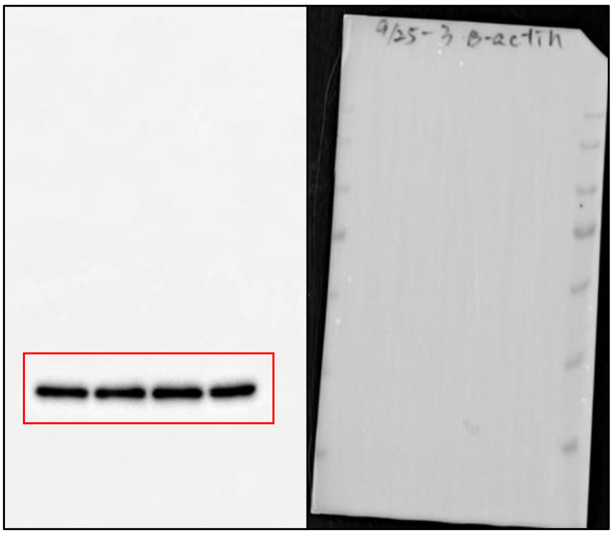

Fig. S1c. p-TRKs

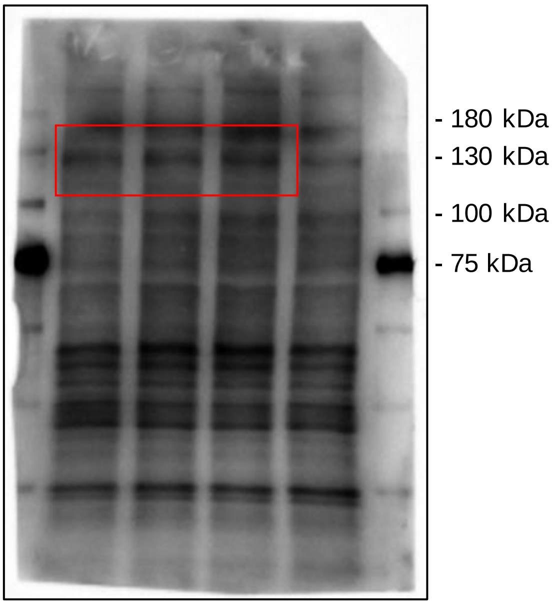

Fig. S1c. β-actin

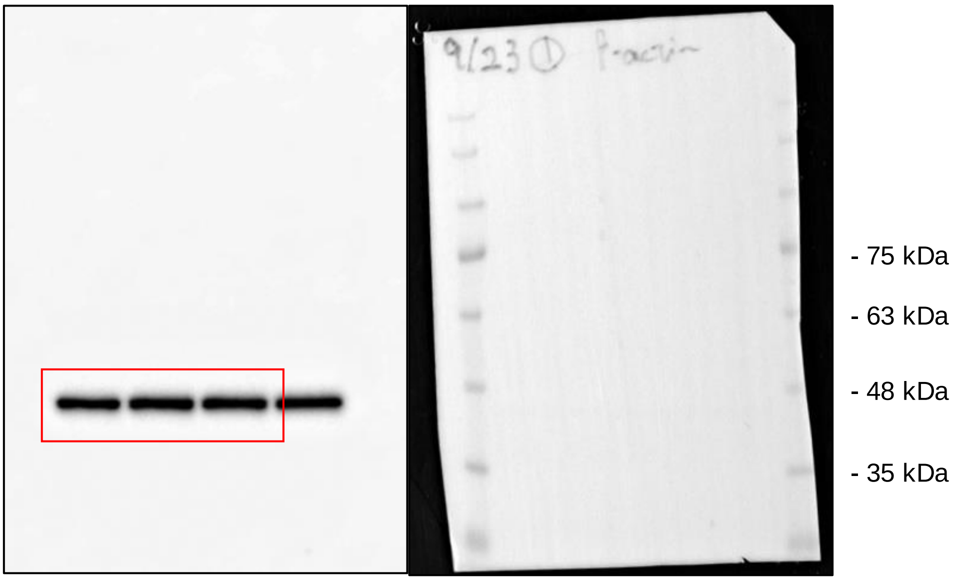

Fig. S2. BDNF

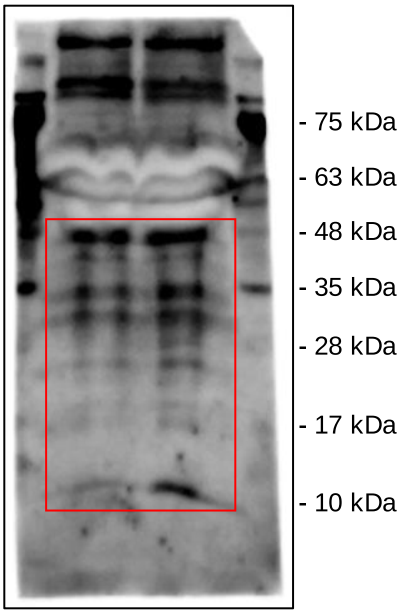

Supplement: Supplementary file 1 — Supplementary Figures. [file 41598_2022_22377_MOESM1_ESM.pdf]
